# Iatrogenic cerebral amyloid angiopathy after red blood cell transfusion?

**DOI:** 10.1111/ene.16366

**Published:** 2024-06-04

**Authors:** Jacopo C. DiFrancesco, Payam Tabaee Damavandi, Janja Pretnar‐Oblak, Senta Frol, Patricia de la Riva Juez, Ines Albajar Gomez, Ulf Jensen‐Kondering

**Affiliations:** ^1^ Department of Neurology Fondazione Istituto di Ricovero e Cura a Carattere Scientifico San Gerardo dei Tintori Monza Italy; ^2^ Department of Neurology Neurocenter of Southern Switzerland, Ente Ospedaliero Cantonale Lugano Switzerland; ^3^ Department of Vascular Neurology, Faculty of Medicine University Medical Centre Ljubljana Ljubljana Slovenia; ^4^ Stroke Unit Donostia University Hospital San Sebastian Spain; ^5^ Department of Neuroradiology University Hospital Schleswig‐Holstein Lübeck Germany

Dear Editor,

Cerebral amyloid angiopathy (CAA) is caused by deposition of amyloid fibrils in cerebral vessels, resulting in hemorrhagic events. Recently, it has been reported that iatrogenic CAA (iCAA) may be transmitted by surgical instruments, cadaveric dura mater grafts, or growth hormones [1]. In a huge registry analysis (>1 million analyzed patients) from two large nationwide cohorts from Sweden and Denmark, Zhao and coworkers provide compelling statistical evidence for possible transmission by red blood cell (RBC) transfusion. By using multiple intracerebral hemorrhages (ICHs) as a proxy for CAA, they demonstrated a more than twofold hazard increase for developing a single ICH after RBC transfusion from donors who subsequently developed multiple ICHs [2]. However, absolute numbers were small; only 26 RBC recipients developed single or multiple ICHs. Because no detailed clinical or neuroradiological information on either donors or the recipients was obtained, it remains uncertain whether the donors harbored and whether the RBC recipients developed CAA.

We commend Kaushik and coworkers on the identification of two patients with probable CAA who were recipients of RBC transfusions in their infancy [3]. Although no information about the donors was obtained and the diagnosis of CAA was made noninvasively, this constitutes a first half‐step towards closing the gap in demonstrating a possible transmission by blood.

The European Iatrogenic Cerebral Amyloid Initiative recently reported on 27 patients with iCAA [4]. Since then, two more patients were identified [5, 6]. In a subset of 11 patients whose data were ad hoc available, we identified five patients who had a history of RBC transfusion, after retrospective chart review and/or patient interviews. In all cases, RBC transfusion coincided with index neurosurgery and/or dura transplant (Table 1). The rate of RBC transfusion was 45% (5/11 patients), exceeding the reported rate of RBC transfusion after traumatic brain injury (36%, 95% confidence interval = 28%–44%) [7] and pediatric brain tumor surgery (25%) [8], the two most common underlying conditions in our cohort.

**TABLE 1:** Demographics of the patients with iCAA.

#	Country	Sex	Neurosurgery	Dura graft	TBI	Age at surgery	RBC transfusion	Age at RBC transfusion	Age at CAA‐related presentation, years	Latency, surgery–presentation, years	Queen Square criteria for iCAA
1	Germany	Male	Yes	Yes	No	3 months	Yes	3 months	35	35	Probable
2	Slovenia	Male	Yes	Yes	Yes	7 years	Yes	7 years	45	38	Possible
3	Slovenia	Female	Yes	Yes	No	9 years	No	NA	46	35	Probable
4	Spain	Female	Yes	Unknown	No	21 years	No	NA	49	28	Possible
5	Spain	Male	Yes	Unknown	Yes	3 years	Yes	3 years	33	30	Possible
6	Spain	Male	Yes	Unknown	No	12 years	Yes	12 years	52	40	Possible
7	Spain	Male	Yes	Unknown	No	21 years	No	NA	60	39	Possible
8	Italy	Male	Yes	Unknown	No	22 years	No	NA	61	39	Probable
9	Italy	Female	Yes	Yes	No	32 years	No	NA	72	40	Probable
10	Italy	Male	Yes	Unknown	No	21 years	No	NA	55	34	Probable
11	Italy	Male	Yes	Yes	Yes	10 years	Yes	10 years	54	44	Probable

Abbreviations: CAA, cerebral amyloid angiopathy; iCAA, iatrogenic CAA; NA, not applicable; RBC, red blood cell; TBI, traumatic brain injury.

However, no hard conclusions can be drawn, considering the high total number of patients (>10,000) who developed ICH after RBC transfusion in the original study. Furthermore, we have to consider missing data due to incomplete documentation. At the very best, they demonstrate that some patients who had an RBC transfusion developed CAA and not just multiple ICHs.

According to the European directive 2002/98/EC, records that allow for traceability of donors shall be kept for 30 years. Donors of RBCs who donated after the introduction of these directive could thus be identified with a reasonable effort. However, patients in our cohort received RBC transfusion before the directive was introduced, or the time limit of record keeping had already passed.

Apart from a new mode of transmission (for a discussion of the current state of knowledge, see the editorial by Greenberg [9]), this also could have wide‐ranging implications for the practice of blood donation. Should donors with CAA or presymptomatic CAA be excluded from donating blood or blood components? How would screening take place? Should serum amyloid levels or magnetic resonance imaging become the standard of care? As pointed out before, more digging is warranted.

## AUTHOR CONTRIBUTIONS

**Jacopo C. Di Francesco:** Writing – review and editing. **Payam Tabaee Damavandi:** Writing – review and editing. **Janja Pretnar‐Oblak:** Writing – review and editing. **Senta Frol:** Writing – review and editing. **Patricia de la Riva Juez:** Writing – review and editing. **Ines Albajar Gomez:** Writing – review and editing. **Ulf Jensen‐Kondering:** Writing – review and editing; writing – original draft.

## CONFLICT OF INTEREST STATEMENT

U.J.‐K. has received an honorarium from Henry Steward Talks. The other authors declare that they do not have a conflict of interest.

## Data Availability

The data that support the findings of this study are available on request from the corresponding author. The data are not publicly available due to privacy or ethical restrictions.
